# Improving the detection, assessment, management and prevention of delirium in palliative care units (the DAMPen-Delirium II study): protocol for an implementation-to-target Type III hybrid effectiveness-implementation cluster randomised-controlled trial, economic evaluation and process evaluation of the Creating Learning Environments for Compassionate Care-Palliative (CLECC-Pal) implementation strategy

**DOI:** 10.1186/s13012-026-01522-2

**Published:** 2026-07-24

**Authors:** Gillian Jackson, Grant Constable, Bronwen Williams, Judith Cohen, Chao Huang, Julie Halliwell, Helen Roberts, Daniel Harman, Paul Taylor, Peter May, Alicia O’Cathain, Annmarie Hosie, Meera Agar, Miriam Johnson, Fliss E. M. Murtagh, Mark Pearson

**Affiliations:** 1 Wolfson Palliative Care Research Centre, Hull York Medical School, University of Hull, Hull, UK; 2 Hull Health Trials Unit, University of Hull, Hull, UK; 3 Institute of Clinical & Applied Health Research, Hull York Medical School, University of Hull, Hull, UK; 4 Public Co-investigator and Chair of Public Advisory Group, Manchester, UK; 5 City Health Care Partnership CIC, Hull, UK; 6 School of Medicine and Population Health, University of Sheffield, Sheffield, UK; 7 Cicely Saunders Institute of Palliative Care, Policy & Rehabilitation, Florence Nightingale Faculty of Nursing, Midwifery & Palliative Care, Kings College London, London, UK; 8 School of Medicine, Trinity College Dublin, Dublin, Ireland; 9 The University of Notre Dame, Darlinghurst, Sydney, Australia; 10 University of Technology Sydney, Sydney, Australia

**Keywords:** Delirium, Palliative care, Hospices, Guideline adherence, Cost-benefit analysis

## Abstract

**Background:**

Delirium affects up to two-thirds of palliative care unit (PCU) inpatients, causing distress for patients, families, and staff, and contributing to complex care needs and increased healthcare resource use. Although evidence-based guidelines exist, their consistent implementation in PCUs is limited, and implementation strategies to support guideline-adherent care have not been rigorously evaluated in this setting. Creating Learning Environments for Compassionate Care-Palliative Delirium (CLECC-Pal Delirium) is a co-designed multi-component implementation strategy to help embed guideline-adherent delirium care within routine PCU practice. This cluster randomised controlled trial (cRCT) evaluates the effectiveness and cost-effectiveness of the implementation strategy using an implementation-to-target design, with embedded economic and process evaluations.

**Methods:**

Adaptive implementation-to-target, Type III hybrid effectiveness-implementation parallel group cRCT across 20 PCUs (10 per arm, ~ 50 patient records per cluster at each timepoint) in the UK, randomised 1:1 to CLECC-Pal Delirium or usual care. Randomisation is stratified by unit size, education provision, and provider type (NHS/Charity). Sites will receive tailored support to achieve predefined implementation-readiness criteria prior to data collection. The primary outcome is the proportion of each admission’s inpatient days affected by delirium (delirium days), measured retrospectively using a validated chart-based method adapted for palliative care. Secondary outcomes include adherence to delirium care guidelines, patient symptom burden, functional status, and incremental cost-effectiveness. The sample size (1200 admission episodes with delirium) provides 92.3% power to detect a 12% absolute reduction in delirium days, based on an assumed intracluster correlation coefficient (ICC) of 0.03 (α = 0.05). Mixed-effects regression models, with clustering at the PCU level, will be employed to analyse the primary outcome, adjusting for stratification factors and other prespecified covariates, and conducted on an intention-to-treat basis. Blinded statisticians and health economists will conduct analyses. The process evaluation, guided by Normalisation Process Theory, will examine implementation fidelity and how, why, and in what contexts the strategy is integrated into practice.

**Discussion:**

This trial will provide evidence on the clinical- and cost-effectiveness of an implementation strategy to improve delirium care in PCUs, strengthening the implementation evidence base in a setting where implementation research is limited.

**Trial registration:**

ISRCTN23678136; registered 14th April 2025.

## Contributions to the literature

• This study protocol addresses multiple implementation-effectiveness knowledge gaps relating to the common, serious and distressing condition of delirium in palliative care units.

• Internationally, it is the first ever national randomised trial to be conducted of a theoretically-informed, co-designed implementation strategy in palliative care units.

• This hybrid effectiveness-implementation trial demonstrates an efficient trial design by using data from clinical records to rigorously test the effectiveness of an implementation strategy for improving clinical outcomes.

• Conducting an implementation-to-target trial in a novel setting will inform future implementation trial design optimisation and efficiency.

## Background

People nearing the end of life have a high risk of delirium [1, 2], a neurocognitive condition of impaired awareness, attention, and cognition [3]. Delirium is highly distressing for patients and families, increases health professionals’ anxiety and stress [1, 4], and leads to poor clinical outcomes and higher care costs [5–7], yet common causes such as infection or dehydration are preventable and/or treatable. Although the significant negative impacts of delirium on care experience, outomes, costs, and workforce are widely-acknowledged, and systematic reviews have identified barriers to implementing delirium management and prevention [8, 9], implementation strategies for guideline-adherent delirium assessment, management and prevention have not been rigorously tested. A recent Cochrane review of non-pharmacological approaches to preventing delirium outside of intensive care concluded that future research should focus on implementation and how intervention components should be tailored for different settings [10]. Addressing this gap requires rigorous evidence about the effectiveness of an implemementation strategy designed to support teams to integrate evidence-based delirium care into routine practice.

The *clinical issue* is that one-third of people cared for in adult in-patient Palliative Care Units (PCUs), such as hospices, have delirium on admission, with a further one-third developing delirium during their stay [2], yet a substantial proportion of delirium risk in this population is attributable to modifiable factors such as medication effects, dehydration, hypoxaemia, and sleep disturbance [1, 2, 11, 12]. In practice, delirium is often underestimated due to hypoactive delirium (where patients are quiet and withdrawn) not being recognised [13]. About half of PCU in-patients are admitted for symptom management and discharged following optimised management, yet an episode of delirium carries a higher risk of subsequent cognitive decline, care home placement or repeat hospital admissions [7]. Delirium causes increased length of hospital stay, staff time, and higher levels of hospital-associated adverse events, leading to higher costs of care (e.g. £349 average cost per palliative care bed day) [14] and additional suffering for patients. Although more evidence is needed, delirium interventions may be cost-effective by preventing and shortening delirium episodes, reducing falls, and improving functional ability [6]. Unplanned hospital admissions are also reduced [15].

Whilst poor outcomes may appear less important for people with shorter prognoses, gaining quality time at home without mental deterioration is extremely important for many. Delirium also jeopardises care in the community, and our Public Advisory Group have confirmed how preserving mental capacity and communicating with loved ones is crucial for decision-making and reducing distress. In short, the impact of delirium and its psychological sequelae for patients, carers, and healthcare staff is under-acknowledged, despite the significance of the distress caused [1, 16, 17].

Guidelines [18, 19] and standards [20] define the *evidence-based clinical intervention* for delirium prevention, timely detection and non-pharmacological management, (with pharmacological interventions if appropriate) [21, 22]. However, implementation of guideline-adherent care is poor, despite the person-centred culture and good staff levels of palliative care units [23]. This leads to persistent poor practice such as routine use of antipsychotics to treat delirium despite evidence to the contrary [24, 25] and low rates of use in PCUs of validated delirium risk-assessment tools such as the 4AT [26, 27]. In PCUs, the complex interactions between patients, family, and clinicians in diverse organisational and cultural contexts [3, 23, 28] require implementation strategies that extend beyond education to include practical and emotional support for staff [3]. 

However, Whilst there have been valuable insights from trials of multi-component delirium interventions (without implementation strategies) in hospital settings, a pilot study and recent protocol for a patient-centred delirium monitoring system in Australian PCUs [29, 30], quality improvement projects in PCUs [31, 32], and a feasibility study in care homes [33], there is *limited evidence about the effectiveness of implemementation strategies* for guideline-adherent delirium care in PCUs. To underpin definitive implementation strategy effectiveness research, in our DAMPen-Delirium feasibility study conducted in the United Kingdom (UK) [12], we: (i) tailored a staff educational and empowerment implementation strategy for use in PCUs (CLECC-Pal Delirium); (ii) confirmed we can reliably collect data to assess both patient and implementation outcomes from patient records; (iii) obtained data to calculate sample size for a national cluster randomised controlled trial; and (iv) showed a signal of benefit (6% reduction in proportion of inpatient days affected by delirium, for those with a delirium episode).

*To realise evidence-based decision-making about implementation strategies*, it is crucial to move from developmental and feasibility work to rigorous comparative studies. Implementation science provides a framework for addressing gaps between evidence and routine practice by testing strategies that support the adoption, integration and sustainment of evidence-based care within real-world settings. In complex care environments such as palliative care units, where care is relational, emotionally demanding and delivered by multidisciplinary teams, implementation strategies must be sensitive to local context and organisational-readiness. This study therefore evaluates CLECC-Pal Delirium as an implementation strategy, using an adaptive implementation-to-target approach that supports teams to achieve readiness for change rather than excluding sites on the basis of baseline capacity.

This study protocol describes an implementation-to-target Type III hybrid effectiveness-implementation [34–36] cluster randomised controlled trial with embedded economic and process evaluation. The trial will assess the effectiveness and cost-effectiveness of an implementation strategy for an intervention with known effectiveness (guideline-adherent delirium assessment, management and prevention) on clinical outcomes, and exploring the mechanisms of implementation at staff and organisational levels across diverse palliative care inpatient unit settings in the United Kingdom.

As the first ever national randomised trial to be conducted of a theoretically-informed, co-designed implementation strategy in palliative care units, the DAMPen-Delirium II study will make a significant contribution to implementation science knowledge and understanding by demonstrating how:

1. A trial of an implementation strategy can be delivered in smaller, non-centralised care settings that are typically unfamiliar with Implementation Science.
2. Implementation Science can deliver an efficient trial design that produces actionable results for decision-makers, by using data from clinical records to rigorously test the effectiveness of an implementation strategy for improving clinical outcomes.
3. Application of an implementation-to-target trial design in a novel setting can inform the optimisation of implementation trial designs that maximise efficiency through only proceeding to full evaluation where implementation-readiness criteria are met.

## Aims and objectives

### Aim

To evaluate the effectiveness and cost-effectiveness of a clinical guideline implementation strategy (CLECC-Pal Delirium) to improve the early detection, management and prevention of delirium among palliative care unit (PCU) in-patients.

### Primary objective

To evaluate the effectiveness of the CLECC-Pal Delirium intervention compared to usual practice to reduce the proportion of inpatient days affected by delirium in PCUs in a fully powered cRCT.

### Secondary objectives

1. To identify adherence to delirium care guidelines (for the detection, assessment, management, and prevention of delirium) at baseline and follow up (staff and cluster level).
2. To explore symptom burden and functional status during inpatient admissions, and their relationship with days affected and not affected by delirium (patient level).
3. To evaluate the cost-effectiveness of CLECC-Pal Delirium compared to usual practice on reducing the proportion of inpatient days affected by delirium in PCUs (trial-level economic analysis across all clusters).
4. To improve understanding of how CLECC-Pal-Delirium could be scaled up across the UK (cluster level process evaluation).

## Methods

This trial comprises three work packages. Work Package 1 involved study set-up, regulatory approvals and site identification. This protocol details Work Package 2: an implementation-to-target Type III Hybrid Effectiveness-Implementation cluster randomised controlled trial, with PCUs as the unit of randomisation, chosen to reflect the team-based delivery of the implementation strategy and minimise contamination between trial arms. Embedded within Work Package 2 is an internal pilot phase to monitor site set-up and early data collection processes, an economic analysis (2b) and a Normalisation Process Theory-informed (NPT) process evaluation (2c) to explore how the strategy is understood, enacted and appraised in PCUs. Work Package 3 consists of knowledge exchange workshops designed to support the application of trial findings to other palliative care settings, such as home-based and care home settings.

**Fig. 1:**
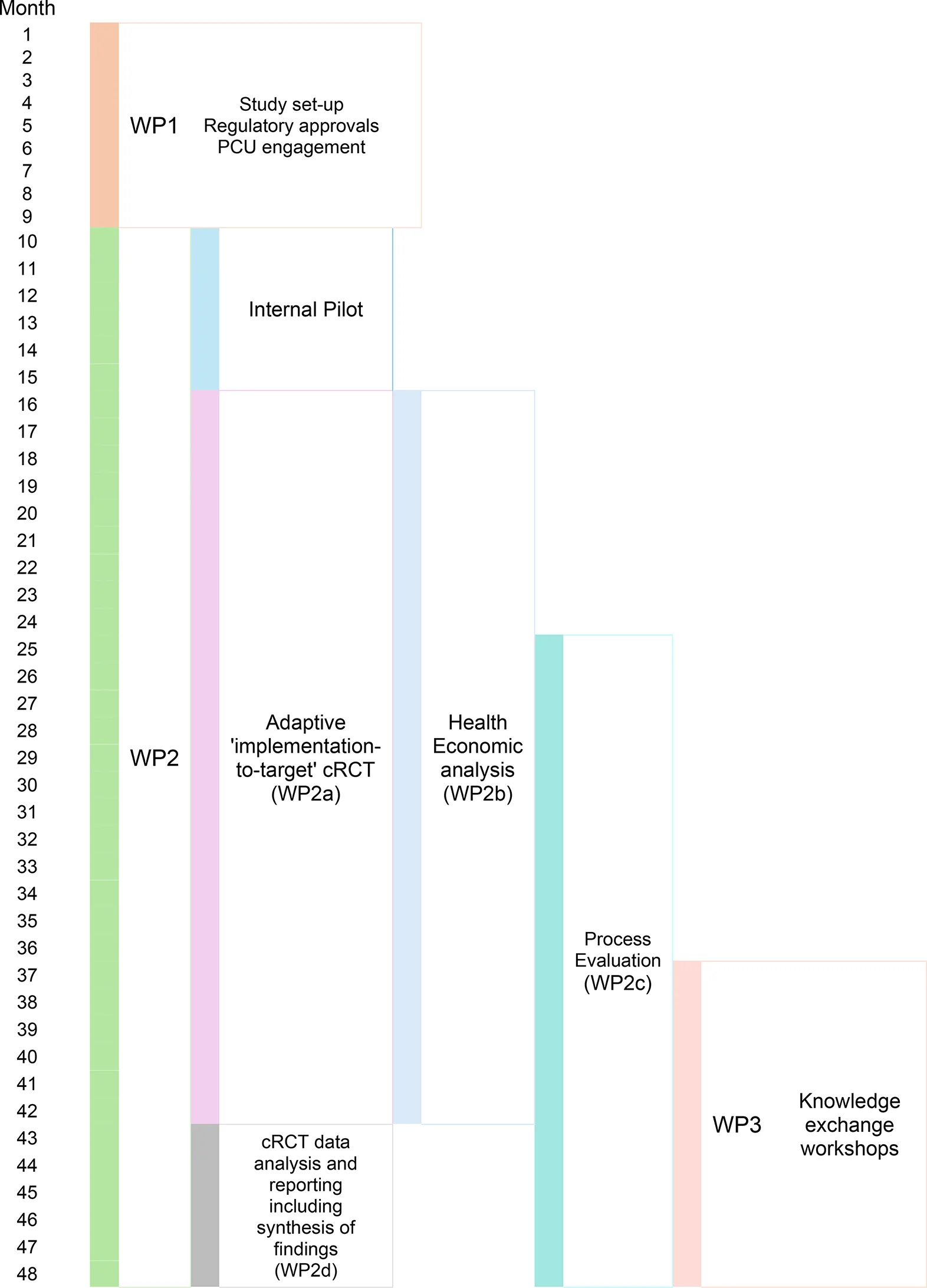
Study timeline (SPIRIT format) showing the sequencing and duration of the three work packages

Work Package 1 and Work Package 3 provide enabling (set-up and engagement) and translational (knowledge exchange) functions respectively. An overview of the study design and work packages is shown in Fig. 1.

### Implementation strategy: CLECC-Pal Delirium

CLECC-Pal Delirium is an implementation strategy adapted from Creating Learning Environments for Compassionate Care (CLECC), designed to support collective learning, reflection, and practice change within care teams [37]. The strategy focuses on how care is organised and delivered by teams, rather than on individual clinician behaviour alone. The CLECC-Pal Delirium implementation strategy is designed to be delivered alongside existing clinical care, rather than replacing routine clinical services within PCUs. Site PCUs randomised to the CLECC-Pal Delirium implementation strategy arm will implement the components of the CLECC-Pal Delirium implementation strategy. The five components of CLECC-Pal Delirium include a study training day, mid-shift cluster discussions, peer observations of practice, reflective discussions, and action learning sets. The CLECC-Pal Delirium intervention aims to create a secure learning environment that delivers new ways of working, supporting implementation of delirium management guidelines and is described in Table 1. A wide range of site PCU staff e.g. healthcare assistants, nurses, allied health professionals (e.g. physiotherapist, OTs), therapy professionals (e.g. music therapists), doctors, volunteers, care managers and executive board members will be expected to take part in the components of CLECC-Pal Delirium as part of their day-to-day practice.

#### Harms

No formal adverse event reporting is planned; unintended effects will be identified through study data collection.

**Table 1:** CLECC-Pal Delirium implementation strategy (TIDieR format) [38]

Component	Why	What	Who, How, Where	When/How often
Study Day	To introduce delirium care principles and the CLECC-Pal Delirium strategy	Presentations, interactive activities, discussions, and planning	All patient-facing staff and volunteers, on-site, led by research team	One day
Mid-shift Cluster Discussions	To facilitate peer learning, reflection, and real-time problem solving	Short focused discussions on delirium cases or concerns	Ward teams during shifts	5 min daily
Peer Observation of Practice	To promote learning from practice and reinforce behavioural change	Observations followed by supportive feedback	Conducted by PCU Delirium Lead	6–10 observations, 5–60 min each
Reflective Discussions	To encourage emotional processing and team cohesion	Facilitated group reflections, case discussions, and narrative sharing	All staff, including temporary or agency staff, facilitated by clinical lead	Frequency determined locally (at least once)
Action Learning Sets	To identify barriers and co-develop solutions to improve delirium care	Team-based action planning focused on implementation challenges	All staff, including senior management, facilitated by clinical lead	Frequency determined locally (at least once)

### Work Package 2a: Adaptive implementation-to-target cRCT

#### Setting and participants

The trial (Work Package 2) will be conducted in 20 PCUs across the United Kingdom (England, Wales and Scotland), selected to reflect variation in charity or NHS hospice, size, and geographical setting to capture diversity in ethnicity and socioeconomic status. Site selection is guided by Firth’s criteria [39], which consider organisational educational capacity and contextual factors that influence implementation potential. Informed by the findings of our feasibility study, sites will be assessed for implementation readiness prior to implementation strategy delivery. The implementation-readiness criteria to be met are:

1. Site local governance approval of rapid delirium assessment tool (e.g. 4AT) for clinical notes.
2. Identified lead for a minimum of three CLECC-Pal Delirium components, including at least one nurse.
3. Expressed PCU management support for using the CLECC-Pal Delirium implementation strategy.

Sites that do not meet these criteria initially will receive additional graduated support as part of the adaptive ‘implementation-to-target’ approach.

#### Eligibility criteria

Cluster participants are PCUs, which serve as the unit of randomisation and the level at which the CLECC-Pal Delirium implementation strategy is introduced. Cluster PCUs: Eligible clusters are adult inpatient palliative care units (PCUs) anywhere in the UK. All clusters are eligible to take part in the trial regardless of implementation readiness for CLECC-Pal Delirium; however, those randomised to the CLECC-Pal Delirium arm must meet the specific readiness criteria. Clusters that do not meet these criteria at first will receive targeted support to achieve readiness before full strategy implementation. These criteria, developed from feasibility study findings [12], include PCU management support for use of CLECC-Pal Delirium, named leads for at least three CLECC-Pal Delirium components (one of whom must be a nurse), and confirmation of local governance arrangements.

Staff: All staff and volunteers with direct patient contact, including healthcare assistants, nurses, allied health professionals, doctors, managers, domestic staff, and catering staff, are eligible to participate in intervention activities. Domestic and catering staff are included because they often interact with patients regularly and may be among the first to notice subtle changes in behaviour or cognition. At intervention sites, all eligible staff are expected to engage with CLECC-Pal Delirium components as part of their routine roles.

Patients: All adult inpatients admitted to participating PCUs during the study opt-out periods are eligible for data collection. Patients who opt-out of data use, either individually or via NHS National opt-out, will be excluded.

A purposive subsample of patients with capacity will be invited to take part in qualitative interviews. Informal carers of eligible patients may also be invited to participate in interviews, either alongside the patient or separately, according to patient preference and consent.

#### Comparator/ usual care

PCUs allocated to the control arm will continue to deliver their usual care, without any additional support or implementation strategies related to delirium. This includes continuation of existing staff practices, training, and clinical policies related to the assessment, prevention, and management of delirium. No elements of the CLECC-Pal Delirium strategy will be introduced at control sites during the study period. To minimise contamination, implementation support and training will be restricted to implementation strategy sites during the trial.

#### Primary outcome (Clinical)

The primary outcome is the proportion of inpatient days affected by delirium during each patient’s PCU admission; readmissions will be considered as separate episodes. This is defined as the number of days with documented delirium divided by the total number of days from admission to discharge or death. Delirium will be identified retrospectively using a validated chart-based method [40], which was adapted and tested for use in the palliative care setting during the feasibility study [12]. This outcome is measured at the patient level (per admission) but will be analysed at the cluster level to account for the cRCT design. Access to identifiable patient records for delirium ascertainment using an opt-out process was approved by Confidentiality Advisory Group (CAG) under Sect.  251.

#### Secondary outcomes (Implementation and clinical)

Secondary outcomes span patient level (symptom burden, functional status), staff and cluster level (guideline adherence), and trial level (cost effectiveness) measures.

- Adherence to delirium care guidelines, including detection, assessment, prevention, and management practices. These will be assessed at baseline and follow-up using predefined care process indicators extracted from patient records.
- Symptom burden and functional status, and their relationship with delirium and non-delirium days. These data will be extracted from clinical records using the Integrated Palliative care Outcome Scale (IPOS) [41] and Australia-modified Karnofsky Performance Status (AKPS) [42], where available.
- Incremental cost-effectiveness ratio quantifying the cost per PCU inpatient day affected by delirium which is avoided.

#### Randomisation

PCUs (the unit of randomisation) will be allocated in a 1:1 ratio to either the CLECC-Pal Delirium intervention or control (usual care) arm. Randomisation will be conducted centrally by the Hull Health Trial Unit (HHTU) data team using a secure, computer-generated allocation sequence implemented via the REDCap Cloud (RCC) randomisation module.

Randomisation by minimisation will be undertaken at the PCU level and stratified based on the following PCU characteristics:

- Size of unit (defined as < 23 monthly admissions = small; ≥23 = large).
- Education provision (categorised as in-unit education centre, external training only, or no training).
- Service type (NHS or charity provider).

**Fig. 2:**
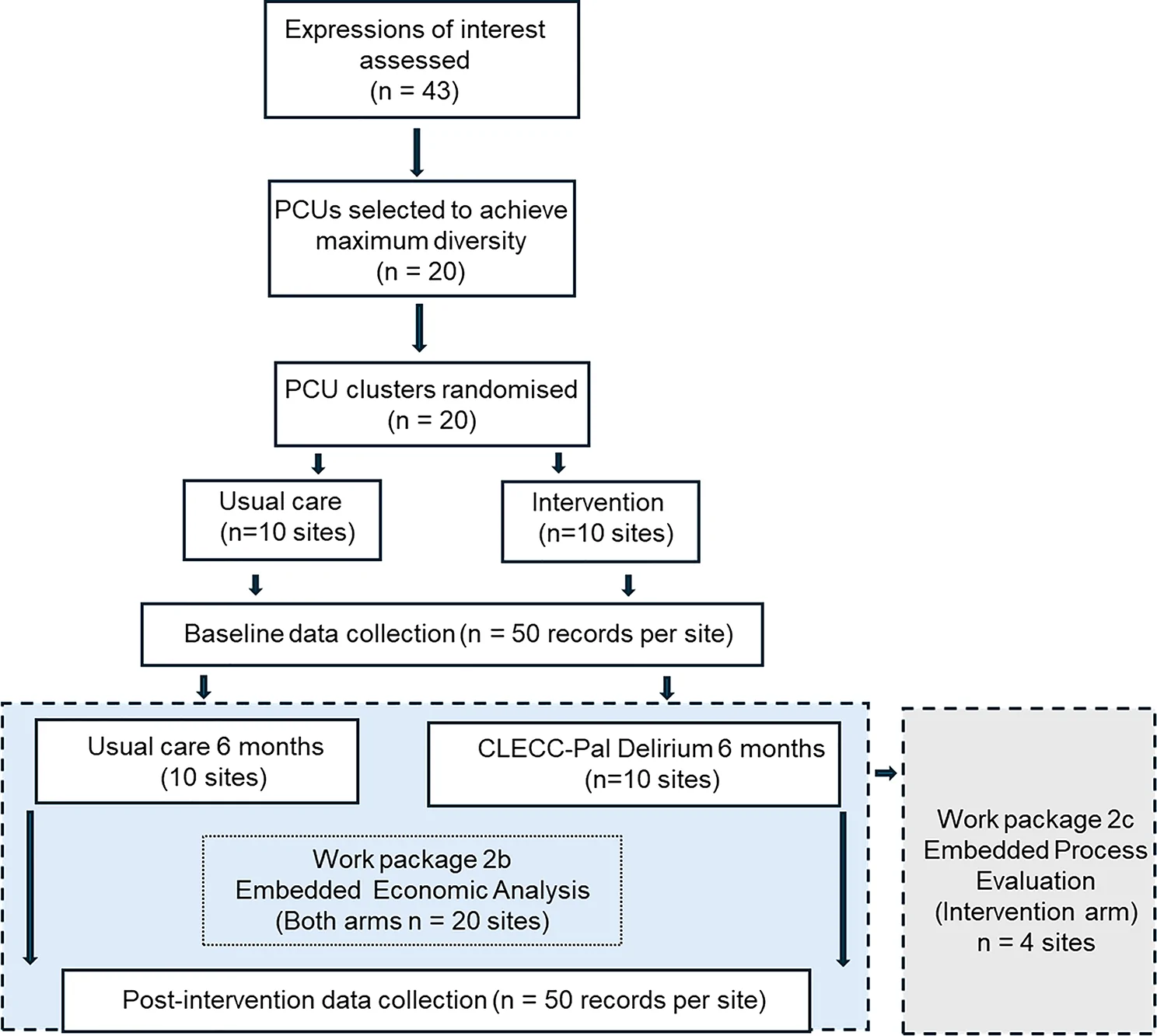
Planned flow of sites and participants through the DAMPen-Delirium II cluster randomised controlled trial, including site selection, randomisation, intervention allocation, and baseline and follow-up data collection

Randomisation will be conducted before any patient data are collected. The allocation sequence will be generated by the HHTU trial manager with the sequence itself concealed; site allocations will be communicated to the study team only as required for operational planning and initiation of intervention delivery. The trial flow, including site selection, randomisation and data collection is summarised in Fig. 2.

#### Sample size

Based on delirium episodes in the previous feasibility study [12], we expect that a minimum of 50 consecutive inpatient records will be required (from each site and at two timepoints) to identify 30 delirium episodes at baseline and 30 episodes at follow-up (total 1200 delirium data records). We will collate data records from PCU inpatients (stopping record identification at each site as soon as 30 delirium episodes have been identified) at both timepoints (baseline and follow-up) from 20 sites to obtain a total of 1200 episodes of delirium (600 baseline and 600 follow-up) to achieve a 92.3% power to detect a 12% reduction of proportion of inpatient days affected by delirium, at 0.05 significance level, assuming an ICC of 0.03. The ICC (0.03) and the average cluster size assumption (~ 50 patient records per cluster/site at each timepoint) are also derived from the previous feasibility study, which provided preliminary estimates for variability and informed the definitive trial design [12].

#### Data collection and blinding

Outcome data (Table 2) will be collected retrospectively from inpatient records using a predefined database. Functional status, symptom burden, and adherence to guideline-based delirium care will be extracted where documented using routine clinical tools (e.g. IPOS, AKPS). Delirium episodes will be identified using the validated chart-based method by Inouye et al., adapted for palliative care settings [40]. This does not require individual consent, as approval for access to confidential patient information was obtained from the Confidentiality Advisory Group (CAG) under Sect.  251. To ensure transparency, all participating PCUs will display a patient information leaflet in admission packs and posters explaining the use of routinely collected data for this study, how confidentiality is protected, and who to contact with concerns. These materials were reviewed and approved as part of the CAG application. Site staff are also briefed about the data collection processes as part of study onboarding and will where possible, make patients and their families aware of the study on or close to admission and signpost them to the study researchers if they require further information.

**Table 2:** Data collection (completed by study researchers)

Primary Endpoint	Measure	How	Timepoints
Proportion of inpatient days affected by delirium in PCUs, in a fully powered cRCT.	For patients who experience delirium during PCU admission, the proportion of inpatient days affected by delirium, calculated as the number of days they experience delirium divided by their total number of inpatient days	Data extraction from • Paper • Medical records e.g. SystmOne/EMIS	Two timepoints: **Baseline** 50 consecutively admitted inpatients who completed their inpatient stay during the time period from display of poster to start of intervention *Note: As this is information entered into the inpatient records on the day of events*,* baseline data can be collected retrospectively at any point during the study* **Follow up** 50 consecutively admitted inpatients who completed their inpatient stay from the beginning of the seventh calendar month after starting the intervention (in the intervention arm) or continuing usual practice (in the control arm)
**Secondary Endpoints**	**Measure**	**How**	**Timepoints**
To identify adherence to delirium care guidelines (for the detection, assessment, management, and prevention of delirium) at baseline and follow up • 4AT screening tool [43] • Delirium risk assessment • Richmond Agitation-Sedation Scale-Palliative version [44] • Clinician-documented diagnosis of delirium • Clinician assessment of cause/reversibility • Use of antipsychotics in relation to documented harmful/distressing behaviour towards self or others • Delirium care plan • Medication review	Present in the records? Yes / No	Data extraction from: • Paper records • Medical records e.g. SystmOne/EMIS	As above
To explore symptom burden and functional status during inpatient admissions • Integrated Palliative care Outcome Scale (IPOS) • Australian-modified Karnofsky Performance Scale (AKPS)	Tool score		
**Demographics and clinical characteristics**	**Measure**		
Age, Sex, Primary medical condition, Ethnicity, Postcode (converted to IMD score at data collection), Date of death (if applicable), Length of stay.	Not applicable		

Due to the nature of the intervention, PCU staff and patients will not be blinded. However, outcome data are collected retrospectively, and the trial statistician and health economist will remain blinded to allocation during analysis. Study researchers extracting delirium data from patient records are not blinded to site allocation due to local data access requirements. To minimise bias, a standardised data extraction protocol is used across all sites, with training, monitoring and audit checks for consistency and accuracy [45, 46]. 

#### Analysis

The primary analysis will assess the difference in the proportion of inpatient days affected by delirium between trial arms, using a mixed-effects model to account for clustering at the PCU level. Analyses will follow the intention-to-treat principle. Models will adjust for stratification variables (unit size, education status, and provider type) and other prespecified covariates. Secondary outcomes will be analysed using appropriate regression models based on outcome type (e.g. linear, logistic), also accounting for clustering. As the data collection is conducted by reviewing the required number of patient notes, potential data missingness will be minimised. If substantial missingness exists in final clinical outcome data, multiple imputation by chained equations (MICE) will be considered, as specified in the final Statistical Analysis Plan (to be finalised prior to completion of data collection and commencement of analysis), with sensitivity analyses conducted to assess the impact of different assumptions.

### Work Package 2b: Economic evaluation

Work Package 2b comprises an embedded economic evaluation conducted alongside the cRCT to assess the cost-effectiveness of the CLECC-Pal Delirium implementation strategy.

#### Data

Following Data Access Request Service (DARS) approval, we will estimate costs (NHS/provider perspective) in two settings: PCU (via routine data) and acute hospital (via linked Hospital Episode Statistics (HES) data) [47], with all data housed and analysed exclusively in the University of Hull’s Data Safe Haven. We will estimate the cost of a day in PCU, stratified by PCU type, using Personal Social Services Research Unit (PSSRU) data [48], differentiating between staffing costs for patients with and without delirium based on staff logs and discussions with clinicians and managers.

We will identify acute hospital admissions via linked HES data and estimate associated costs using NHS reference costs adjusted for Healthcare Resource Group (HRG) codes, delirium status, and length of stay. We will also adjust for the additional cost associated with an acute hospital admission ending in death.

We will estimate the resources associated with the CLECC-Pal Delirium strategy through review of staff activity and discussions with clinicians and managers. All costs will be reported in GBP and valued in the most recent price year available at the time of analysis, with earlier costs inflated using the health section of the consumer price index [49]. 

#### Analysis

The primary economic evaluation will adopt a within-trial time horizon corresponding to the index PCU admission. This reflects the short-term nature of delirium outcomes and the availability of resource-use data during admission. If important downstream consequences of delirium (e.g. hospital readmissions or mortality differences) are observed, we will explore extending the analysis using a short decision-analytic model. We will estimate the incremental effectiveness relative to usual care using the primary outcome (PCU inpatient days affected by delirium). We have chosen cost-effectiveness analysis over cost-utility analysis in the context of practical and ethical challenges in primary data collection of generic health related quality of life (HRQoL) data in our population and study design [50]. Cost-effectiveness will be summarised using the incremental cost-effectiveness ratio (ICER), defined as the difference in mean costs between groups divided by the difference in mean effectiveness (inpatient days affected by delirium avoided). In secondary analyses, we will explore relationships between IPOS [41] and AKPS [42] scores with delirium and non-delirium days at sites where these data are collected and explore the scope for using these in cost-effectiveness analyses, thus investigating if incorporating a wider set of HRQoL domains affects our interpretation and conclusions.

Prior to conducting primary analysis, we will examine: (i) baseline differences on characteristics associated with outcome and where necessary control for baseline variables in analysis; and (ii) skew, kurtosis and heteroscedasticity in the cost data and fit an appropriate (most likely, nonlinear) model [51]. We will address missing outcome data using multiple imputation. We will bootstrap each set of regressions with 1000 replications. If data characteristics require it, we will account for correlated costs and effects using seemingly unrelated regressions. Recognising the uncertainty associated specifically with a cRCT, we will employ a stratified two-stage nonparametric bootstrap resampling procedure for clustered data [52]. For the purposes of cost-effective analyses, each day affected by delirium will be treated as a ‘whole (24 hour) day’. Results will be presented on cost-effectiveness planes and summarised using cost-effectiveness acceptability curves. As the within-trial time horizon is less than one year, discounting will not be applied. If modelling beyond one year is undertaken, costs and outcomes will be discounted in line with NICE guidance.

### Work Package 2c: Process evaluation

Work Package 2c comprises an embedded process evaluation, guided by Normalisation Process Theory (NPT) [53], to examine how the CLECC-Pal Delirium implementation strategy is understood, enacted, and appraised within participating palliative care units. The process evaluation will be conducted in a purposively selected subset of four intervention PCUs to enable in-depth evaluation of implementation processes.

#### Fidelity

Fidelity will be assessed using routine monitoring data collected from intervention sites. This will include the proportion of eligible staff participating in each component of the CLECC-Pal Delirium strategy, reported overall and by staff group.

#### Qualitative interviews

Semi-structured interviews will be conducted with both staff and patients/informal carers at four purposively sampled intervention PCUs. Sites will be selected to reflect variation in PCU type, size, population served, and previous research participation. Purposive sampling will also ensure inclusion of patients who have experienced delirium and those who have not, as the intervention addresses both prevention and management. Within each selected site, 6–10 staff and 6–10 patients or informal carers will be invited to take part.

#### Eligibility and exclusion criteria

Eligible participants will include PCU staff with patient contact or in management roles, inpatients with capacity to consent, and informal carers. Patients lacking capacity at the point of interview will not be interviewed directly, but their informal carers will still be eligible for interview. Staff without patient contact (excluding management roles) will also be excluded.

Staff interviews will explore engagement with CLECC-Pal Delirium using the four NPT constructs:

- Coherence – how staff understand the intervention and its purpose.
- Cognitive participation – how staff commit to and engage with the intervention.
- Collective action – how the intervention is enacted in daily work.
- Reflexive monitoring – how staff appraise its impact and usefulness.

Patient and informal carer interviews will explore experiences of delirium care during PCU admission, including both prevention and management strategies.

#### Ethical considerations

Following assessments of patient decision-making capacity, the necessity (or otherwise) to be approached whilst an informal carer is present, and the absence (at time of approach) of delirium, potential patient and carer participants will be identified and approached by the site Principal Investigator who will verbally explain the study and provide written information (developed with feedback from members of our Public Advisory Group to improve clarity of language and presentation). Patients and carers will be given sufficient time to consider participation, and written informed consent will be obtained before any interviews take place. Patients and carers may choose telephone, video, or face-to-face interviews and will be given options for joint or separate interviews depending on their preference. Interpreters and translated materials will be available where needed.

The researcher undertaking the interviews will listen to patients and informal carers sensitively, and allow breaks or withdrawal from the interview if they wish. It will not be the researcher’s role to offer any counselling, but if they are concerned about distress during or after the interview, they will (with the interviewee’s permission) pass their concerns on to the PCU team (with whom we shall work to ensure that clear safeguarding policies and reporting mechanisms are in place). As repeated exposure to issues raised in interviews may be distressing, the work package Lead will meet regularly with the researcher to debrief about their experiences and feelings, referring to clinical members of the research team if required.

#### Analysis

Qualitative data will be generated through semi-structured interviews with staff and patients/informal carers at four purposively sampled intervention PCUs, chosen to capture variation in type, size, population served, and previous research participation (i.e. prior involvement in clinical studies and familiarity with research processes). Interviewers will not be blinded to allocation as all sites are in the intervention arm; to minimise bias and enhance transparency, interviewers will use topic guides that allow for flexibility for site-specific issues, supported by reflexive field notes, peer debriefing, and a documented audit trail of analytic decisions.

Interview data will be transcribed by an approved third party and stored anonymously. Anonymised transcripts will be analysed using the Framework approach [54]. 

We have successfully used the Framework approach for analysing the qualitative data in previous process evaluations. NPT will form part of the thematic framework in the second step of the Framework approach for the staff interviews. Data from early interviews (about five patient/carer interviews, five staff interviews) will be co-analysed with our public co-applicant. We will analyse the data from different sources separately (fidelity, staff interviews and patient/carer interviews) before triangulating them using a joint display based on the Triangulation Protocol [55]. To explore variation in context and implementation, we will use quantitative implementation data from all intervention PCUs (*n* = 10), alongside in-depth qualitative and quantitative data from the four purposively sampled intervention PCUs. Data from the process evaluation will be analysed independently before being triangulated with clinical and economic findings using joint displays to inform interpretation of trial outcomes and understand how implementation processes influenced results [56, 57]. 

### Work Package 3: Knowledge exchange workshops with public and professional stakeholders

Work Package 3 (months 37–48) will focus on knowledge exchange and adaption of the CLECC-Pal Delirium implementation strategy for use across a range of care settings. This will be achieved through a series of four co-designed, online workshops involving carers, service managers, health and care professionals, and commissioners. Drawing on principles of experience-based co-design [58], the workshops will share emerging study findings, explore their implications for practice, and support stakeholders to collaboratively identify and implement context-specific adaptations in settings such as day care, care homes, and people’s homes. Workshop content will be developed iteratively with public involvement co-applicant and Public Advisory Group. The aim being to enable flexible, stakeholder-informed discussion and action planning to support implementation within diverse care contexts.

## Public and patient involvement (PPI)

Public and patient involvement (PPI) has been integral to the design, delivery, and oversight of this trial. During the DAMPen-D feasibility study, a group of four public contributors, one of whom was a co-applicant, played a central role in the experience-based co-design of the CLECC-Pal Delirium intervention. One member of this group also chaired the study’s independent steering committee. This same group will be retained for the current trial, and membership has been expanded to eight individuals, including a new co-applicant who chairs the Public Advisory Group.

The Public Advisory Group will meet regularly and will contribute to all phases of study development, including the refinement of outcome measures, implementation processes, and participant-facing materials. Two additional public contributors sit on the independent trial steering committee. The group will work closely with the research team on transparency and data governance, including co-developing the opt-out poster and patient information materials used at recruitment sites. A regular newsletter will be distributed to all public contributors to ensure ongoing communication and engagement throughout the study.

## Discussion

Delirium remains a highly prevalent and distressing condition among people receiving specialist palliative care, yet implementation of evidence-based guidelines for its prevention and management is inconsistent across palliative care units [9]. This study will address this gap through a cluster randomised controlled trial of a co-designed implementation strategy, CLECC-Pal Delirium, adapted from CLECC, originally developed and validated in acute hospital settings [37] and further tailored for palliative care through our feasibility study [12]. 

A key strength of this trial is its embedded process evaluation, guided by NPT [53], which will provide insight into how, why, and in what contexts the implementation strategy becomes integrated into routine practice. In parallel, the economic evaluation will inform whether CLECC-Pal Delirium offers good value for money when used to reduce delirium and its associated healthcare costs in end-of-life care [6]. 

The trial design reflects the complexity of healthcare implementation by incorporating an implementation-to-target approach, fidelity monitoring, and stakeholder involvement across all levels of the palliative care system. Inclusion of staff from all disciplines, including those often under-recognised, such as domestic and catering staff, is consistent with the CLECC-Pal Delirium model’s focus on collective learning and behavioural change. The choice of pragmatic outcomes including the proportion of inpatient days affected by delirium supports real-world applicability and scalability [59]. 

The study also anticipates operational challenges common in palliative care implementation research. These include site-level variation in governance structures, staffing capacity, and organisational readiness [12]. To address this, the design incorporates an “implementation-to-target” approach, providing additional support to sites that do not initially meet readiness criteria. Maintaining fidelity to the CLECC-Pal Delirium strategy in diverse real-world settings, while balancing the need for local adaptation, presents an ongoing complexity that the process evaluation is specifically structured to explore. In addition, retrospective data extraction on delirium may be affected by variability in documentation quality [60], and mechanisms are in place to monitor and respond to this during the trial.

Findings from this study will contribute to a limited but growing body of rigorous implementation research in palliative care and will offer much-needed guidance on adapting and scaling multi-component implementation strategies in end-of-life settings. Although this trial is conducted in the UK, findings will be relevant to PCUs across the UK and potentially transferrable to similar healthcare settings internationally.

## Data Availability

No datasets were generated or analysed during the current study.

## Declarations

### Dissemination

Findings will be disseminated through peer-reviewed publications, conference presentation, and relevant stakeholder networks. Results will be shared with participating sites, and where appropriate, participants, and reported in the trial registry.

## Abbreviations

AKPS: Australian -modified Karnofsky Performance Scale
CAG: Confidentiality Advisory Group
CLECC-PAL: Delirium Creating Learning Environments for Compassionate Care-Palliative
cRCT: Cluster Randomised Controlled Trial
DAMPen-D: Detection, Assessment, Management, Prevention of Delirium
DARS: Data Access Request Service
EBCD: Experience Based Co-Design
HES: Hospital Episode Statistics
HHTU: Hull Health Trials Unit
HRA: Health Research Authority
HRG: Healthcare Resource Group
HRQoL: Health Related Quality of Life
HSDR: Health & Social Care Delivery Research
ICC: Intraclass Correlation Coefficient
IMD: Index of Multiple Deprivation
IPOS: Integrated Palliative Outcome Scale
ISRCTN: International Standard Randomised Controlled Trial Number
NHS: National Health Service
NICE: National Institute of Clinical Excellence
NIHR: National Institute for Health & Care Research
NPT: Normalisation Process Theory
PCU: Palliative Care Unit
PPI: Patient Public Involvement
PSSCU: Personal Social Services Research
RCC: RedCap Cloud
REC: Research Ethics Committee
REC: Research Ethics Committee
SPIRIT: Standard Protocol Items: Recommendations for Interventional Trials
UK: United Kingdom Declarations
